# Supplementary material for: The Role of Zn Substitution in Improving the Electrical Properties of CuI Thin Films and Optoelectronic Performance of CuI MSM Photodetectors
Source: Materials (Basel). 2022 Nov 17;15(22):8145. doi: 10.3390/ma15228145 (PMC9694342; doi:10.3390/ma15228145)
Supplement: Supplementary file 1 [file materials-15-08145-s001.zip › materials-2014289-supplementary.pdf]

materials

MDPI

Supplementary data

The Role of Zn Substitution in Improving the Electrical Properties of CuI Thin Films and Optoelectronic Performance of CuI MSM Photodetectors

Chien-Yie Tsay [1,*], Yun-Chi Chen [1], Hsuan-Meng Tsai [1], Phongsaphak Sittimart [2] and Tsuyoshi Yoshitake [2]

[1] Department of Materials Science and Engineering, Feng Chia University, Taichung 40724, Taiwan
[2] Department of Advanced Energy Science and Engineering, Kyushu University, Fukuoka 816-8580, Japan
* Correspondence: cytsay@mail.fcu.edu.tw; Tel.: +886-4-2451-7250 (ext. 5312)

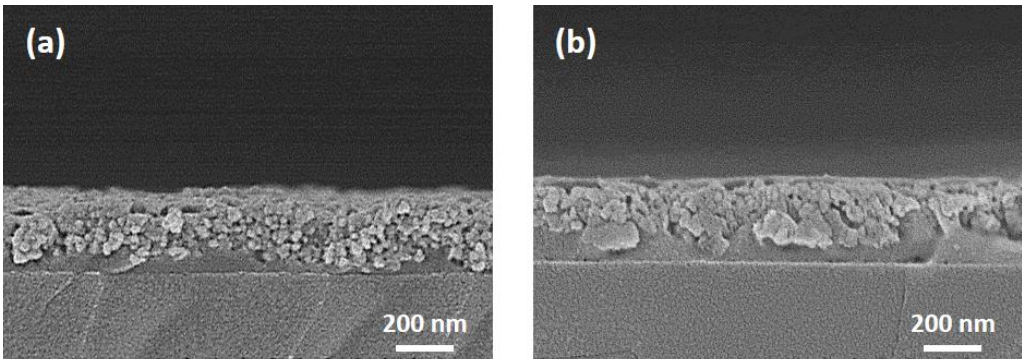

Figure S1. Cross-sectional view FE-SEM micrographs of the pure CuI (**a**) and 8 at% Zn (**b**) substituted CuI thin films on glass substrate.

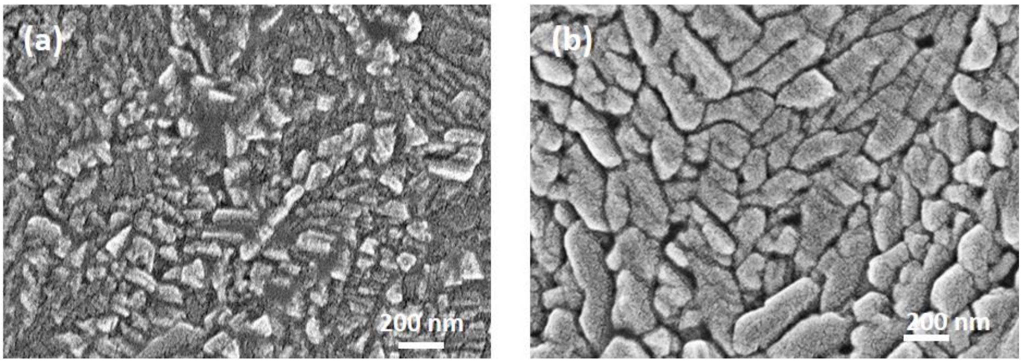

Figure S2. Plane-view FE-SEM micrographs of the pure CuI (**a**) and 8 at% Zn (**b**) substituted CuI thin films.

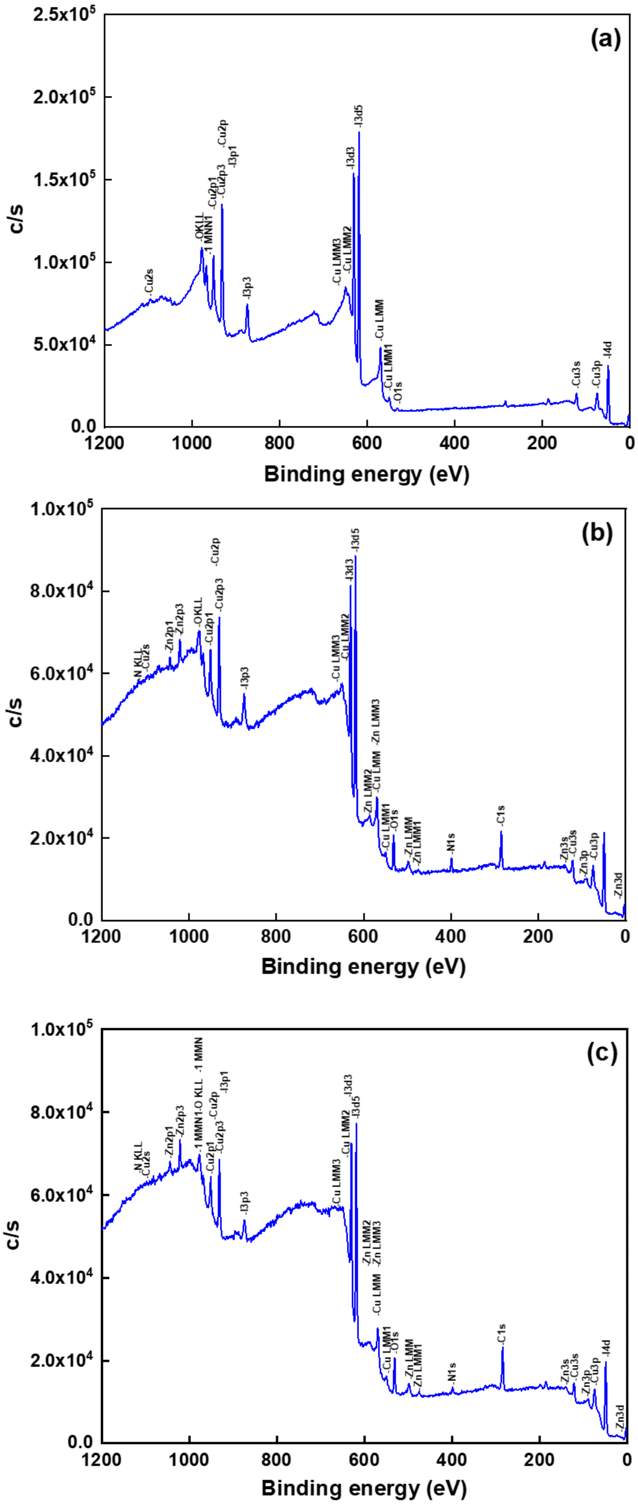
(a)
(b)
(c)
c/s
Binding energy (eV)
1200
1000
800
600
400
200
0
2.5x10^5
2.0x10^5
1.5x10^5
1.0x10^5
5.0x10^4
0.0
1.0x10^5
8.0x10^4
6.0x10^4
4.0x10^4
2.0x10^4
Cu2p
I3d5
I3d3
I3p3
Cu LMM
O1s
Cu3s
Cu3p
I4d
C1s
N1s
Zn3d

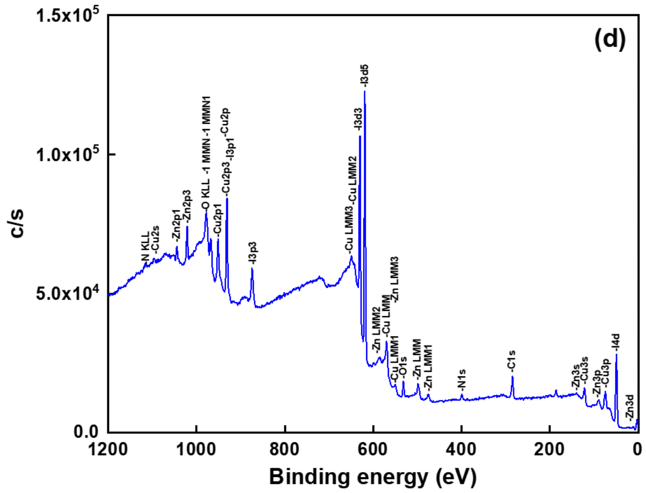

Figure S3. XPS survey spectra of CuI-based thin films: (**a**) 0 at%, (**b**) 4 at%, (**c**) 8 at%, and (**d**) 12 at% Zn substitution.
